# TEVAR and periscope graft technique to treatment of huge aneurysm of aortic isthmus: Case report

**DOI:** 10.1016/j.ijscr.2021.106129

**Published:** 2021-06-19

**Authors:** E. Dinoto, F. Ferlito, M.A. La Marca, D. Pakeliani, G. Bajardi, F. Pecoraro

**Affiliations:** a Vascular Surgery Unit - AOmUP Policlinico ‘P. Giaccone’, Palermo, Italy; b Department of Surgical, Oncological and Oral Sciences, University of Palermo, Italy; c Vascular Surgery Unit, Ospedali Riuniti Villa Sofia-Cervello, Palermo, Italy

**Keywords:** Left subclavian artery revascularization, Staged endovascular aortic repair, Thoracic aortic aneurysm, Zone 2 TEVAR, Periscope graft technique

## Abstract

**Introduction:**

Thoracic endovascular aortic repair (TEVAR) has revolutionized the treatment of thoracic aortic aneurysms. Innovative techniques as chimney and periscope grafts can improve the outcomes of procedure. Herein, we report a case in emergency of huge Thoracic aortic aneurism.

**Presentation of case:**

An 86-year-old male with hypertension, diabetes mellitus, was referred to our hospital for chest pain. CT-angiography showed a huge aneurysm of aortic isthmus with signs of rupture. The patient was considered unfit for open surgery and an endovascular approach was chosen. This patient underwent endovascular repair with TEVAR, using the periscope graft technique to preserve patency in left subclavian artery (LSA).

**Discussion:**

Symptomatic ischemia from LSA coverage has been reported to occur in only a modest 6–10% of patients and is often sacrificed with impunity given coverage rates between 10 and 50%. In this case reported the lack of revascularization of LSA increased the risk of neurological manifestations or stroke. Periscope technique is feasible and safe to maintain perfusion to the subclavian artery, with a 93% primary patency at 2 years.

**Conclusions:**

Our experience using TEVAR with periscope graft technique as solution to address thoracic aneurysm of aortic isthmus was feasible and safe.

## Highlights

• Innovative techniques as chimney and periscope grafts can improve the outcomes of TEVAR.
• Subclavian Artery is important to vascularization of upper arm, vertebro-basilar circulation and medullar vascularization.
• Maintenance of blood flow to the LSA is recommended because it has been shown to prevent paraplegia in TEVAR procedures.
• Periscope technique is feasible and safe to maintain perfusion to the subclavian artery.

## 1 Introduction

Thoracic endovascular aortic repair (TEVAR) has revolutionized the treatment of thoracic aortic aneurysms [1]. The supra-aortic branches limit the proximal extension of the stent-graft. Fenestrated and branched devices have been introduced with promising results in the elective setting to overcome such limitations [2]. In alternative, chimney and periscope grafts have been reported, but experience and follow-up are generally very limited.

Herein, we report a case of huge Thoracic aortic aneurism in complicated patient in emergency where we used a periscope technique to save left subclavian artery (LSA) in patient with right vertebral artery hypoplasia.

This work has been written in accordance with the SCARE criteria [3].

## 2 Case report

An 86-year-old male with hypertension, diabetes mellitus, was referred to our hospital for chest pain. His past medical history includes COPD (Gold D), ischemic heart disease.

**Fig. 1:**
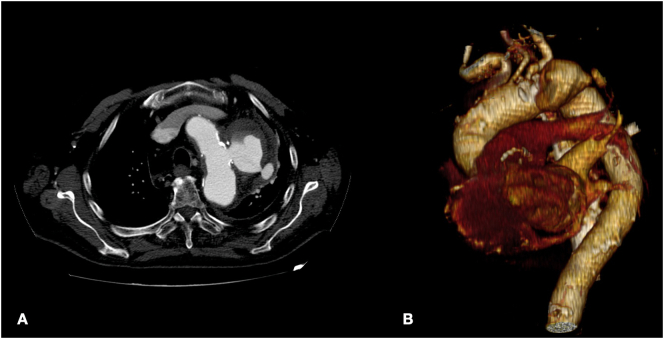
Preoperative CT Angiography (A) and 3-dimensional volume rendering (B) showing aneurism of aortic isthmus.

CT-angiography showed a huge aneurysm of aortic isthmus with signs of rupture. The aneurysm sac measured approximately 7 × 5.5 × 6.5 cm (Fig. 1). The patient was considered to be a poor candidate for open surgery due to significative comorbilities and severe clinical situation and an endovascular approach was chosen.

**Fig. 2:**
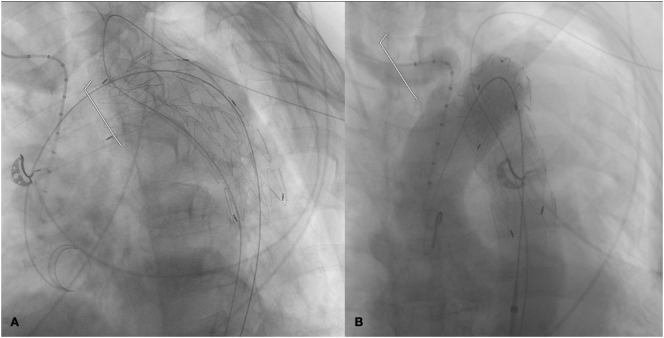
Intraoperative angiography after release of endoprosthesis (A) and final outcome (B).

After general anesthesia and systemic heparinization (ACT >250 s), a right surgical common femoral artery (CFA), a left CFA and bilateral percutaneous brachial artery (BA) accesses were gained. An adequate proximal landing zone was identified in zone 2 Hishimaru aortic arch. In consideration of right vertebral artery hypoplasia, we chose to save left subclavian artery with periscope graft. To maintain LSA perfusion a periscope was planned. Thus, a “through and through wire” from the left CFA to the left BA (bodyfloss technique) [4] was constructed in consideration of the arch anatomy and to create a stable platform for the stent-graft advancement and deployment. A viabahn 10 × 150 cm (W. L. Gore & Associates, Flagstaff, Ariz, USA) was released inside the left subclavian artery with periscope technique. A 38 × 154 cm Bolton aortic endograft (Terumo Aortic, Sunrise, Florida, USA) was placed and deployed in zone 2 aortic to address the aortic isthmus disease. At this stage, the Viabahn covered stent (W.L. Gore & Associates, Flagstaff, Ariz, USA) was deployed. A kissing ballooning of both the Bolton (Terumo) thoracic stent-graft and the LSA periscope was performed. The control angiography confirmed the adequate proximal sealing, the absence of leakages and the maintained LSA patency (Fig. 2).

**Fig. 3:**
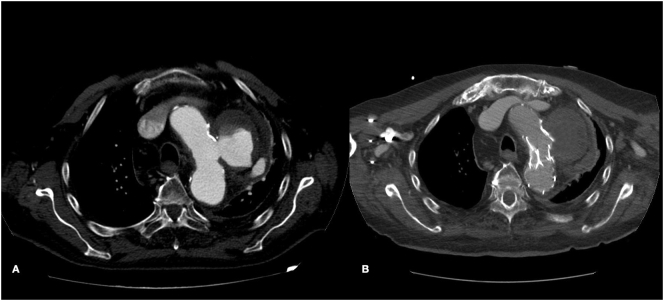
Preoperative CT Angiography (A) and postoperative CT Angiography (B).

**Fig. 4:**
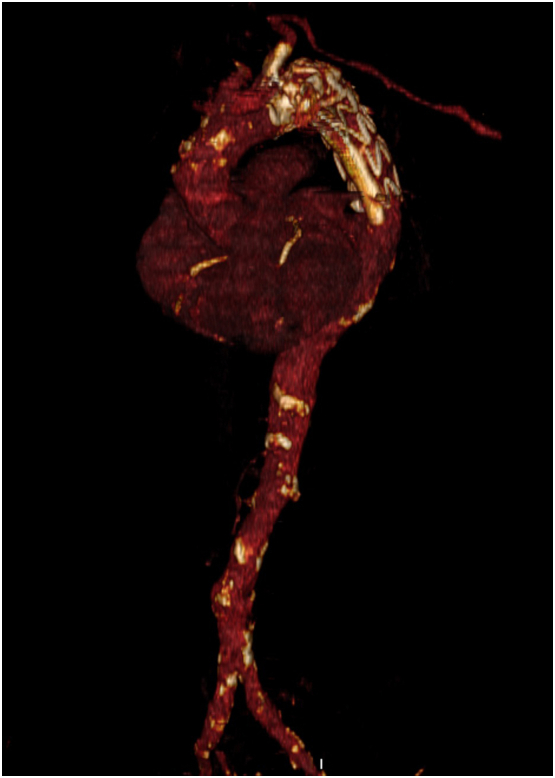
Postoperative CT Angiography 3-dimensional volume rendering showing TEVAR plus periscope graft and patency of left subclavian artery.

After procedure the patient was transferred to the intensive care unit (ICU) for monitoring of vital functions where the extubation was carried after 12 h with no complications or signs of spinal cord ischemia (SCI) (Fig. 3). On the second postoperative day, the patient was transferred from ICU and discharged at home after seven days (Fig. 4).

## 3 Discussion

Subclavian Artery is important to vascularization of upper arm, vertebro-basilar circulation and medullar vascularization. It is common for grafts to require deployment across the origin of the great vessels to obtain proximal seal, thus potentially compromising upper extremity/cerebrovascular perfusion [5]. However, symptomatic ischemia from LSA coverage has been reported to occur in only a modest 6–10% of patients and is often sacrificed with impunity given coverage rates between 10 and 50% [6]. This is because of multiple collaterals beyond the LSA origin, notably retrograde flow from the left vertebral artery, the occipital branch of the external carotid artery, and the superior thyroid artery [7]. Maintenance of blood flow to the LSA is recommended by the Society for Vascular Surgery Practice Guidelines because it has been shown to be an important measure to prevent paraplegia in TEVAR procedures [8]. A TEVAR procedure involving the origin of the LSA is generally managed with: 1) no revascularization; 2) revascularization with open methods; or 3) endovascular revascularization [9]. In case reported, the lack of revascularization of LSA increased the risk of neurological manifestations as dizziness, visual disturbances, stroke due to failure perfusion of the central nervous system [10]. An open solution (Extrathoracic carotid-to-subclavian artery bypass) was excluded and an endovascular approach was only option. Recently, the chimney endograft technique has been reported to be a feasible endovascular approach to maintain LSA flow [4,11]. However, the chimney endograft might destabilize the proximal landing zone of the aortic stent-graft and has been suspected of increasing the risk of type Ia endoleak [12]. Periscope technique is feasible and safe to maintain perfusion to the subclavian artery, with a 93% primary patency at 2 years [13]. The Periscope graft configuration does not interfere with the proximal landing zone of the aortic stent-graft, and the gutters in a periscope configuration are generally longer in comparison to a chimney. This longer sealing zone could reduce the risk of type I endoleak [14].

## 4 Conclusions

This experience using TEVAR with periscope graft technique as solution to address thoracic aneurysm of aortic isthmus was feasible. Our limited experience shows that the use of the periscope endograft technique to maintain perfusion to the LSA is a safe method. The technique is an alternative to open bypass surgery and standard chimney endografts. This solution represents a rapid and less invasive approach, useful in emergency.

## Provenance and peer review

Not commissioned, externally peer-reviewed.

## Funding

None.

## Ethical approval

Not applicable.

## Consent

Not applicable.

## Author contribution

Ettore Dinoto: study concept, design, data collection, data analysis, interpretation, writing the paper, final approval of the version to be submitted, guarantor.

Felice Pecoraro: study concept, design, data collection, data analysis, interpretation, writing the paper, final approval of the version to be submitted.

Ferlito Francesca: study concept, design, data collection, data analysis, interpretation, final approval of the version to be submitted.

La Marca Manfredi Agostino: study concept, design, data collection, final approval of the version to be submitted.

Pakeliani David: study concept, design, data collection, final approval of the version to be submitted.

Guido Bajardi: study concept, design, data collection, data analysis, interpretation, final approval of the version to be submitted.

## Registration of research studies

Not applicable.

## Guarantor

The Guarantor is the one or more people who accept full responsibility for the work and/or the conduct of the study, had access to the data, and controlled the decision to publish.

## Declaration of competing interest

The authors have no ethical conflicts to disclose.

## References

[1] van der Zee C.P., van der Laan M.J., Dinoto E., Tielliu I., Zeebregts C.J., Vainas T. (2019 Feb). A different angle in through-and-through body wires in difficult aortic arch stent-graft placement. J. Cardiovasc. Surg..

[2] Spear R., Haulon S., Ohki T., Tsilimparis N., Kanaoka Y., Milne C.P. (2016). Editor’s choice e subsequent results for arch aneurysm repair with inner branched endografts. Eur. J. Vasc. Endovasc. Surg..

[3] Agha R.A., Franchi T., Sohrabi C., Mathew G., for the SCARE Group (2020). The SCARE 2020 guideline: updating consensus Surgical CAse REport (SCARE) guidelines. Int. J. Surg..

[4] Dinoto E., Pecoraro F., Farina A., Viscardi A., Bajardi G. (2020). Simultaneous endovascular treatment of synchronous symptomatic acute type B aortic dissection and large infrarenal aortic aneurysm. Technical tips and case report. Int. J. Surg. Case Rep..

[5] Dunning J., Martin J.E., Shennib H., Cheng D.C. (2008). Is it safe to cover the left subclavian artery when placing an endovascular stent in the descending thoracic aorta?. Interact. Cardiovasc. Thorac. Surg..

[6] Rizvi A.Z., Murad M.H., Fairman R.M., Erwin P.J., Montori V.M. (2009). The effect of left subclavian artery coverage on morbidity and mortality in patients undergoing endovascular thoracic aortic interventions: a systematic review and meta-analysis. J. Vasc. Surg..

[7] Kim K.G., Grieff A.N., Rahimi S. (2021 Apr 15). Complex endovascular repair of type B aortic dissection and predicting left arm ischemia: a case report. J. Med. Case Rep..

[8] Matsumura J.S., Lee W.A., Mitchell R.S., Farber M.A., Murad M.H., Lumsden A.B., Greenberg R.K., Safi H.J., Fairman R.M., Society for Vascular Surgery (2009 Nov). The Society for Vascular Surgery Practice Guidelines: management of the left subclavian artery with thoracic endovascular aortic repair. J. Vasc. Surg..

[9] Chait J.D., Alsheekh A., Hingorani A.P., Singh N., Marks N.A., Ascher E. (2021). Partial subclavian artery coverage in TEVAR patients for acute type B aortic dissections: an alternative solution. J. Cardiovasc. Surg..

[10] Dinoto E., Pecoraro F., Mirabella D., Ferlito F., Farina A., Lo Biundo N., Conti P., Bajardi G. (2020). Endovascular treatment with drug-eluting balloon for severe subclavian artery stenosis involving the origin of the vertebral artery. Transl. Med. UniSa..

[11] Dinoto E., Ferlito F., Mirabella D., Tortomasi G., Bajardi G., Pecoraro F. (2021). Type 1A endoleak detachable coil embolization after endovascular aneurysm sealing: case report. Int. J. Surg. Case Rep..

[12] Hogendoorn W., Schlösser F.J., Moll F.L., Sumpio B.E., Muhs B.E. (2013). Thoracic endovascular aortic repair with the chimney graft technique. J. Vasc. Surg..

[13] Lachat M., Mayer D., Pfammatter T., Criado F.J., Rancic Z., Larzon T., Veith F.J., Pecoraro F. (2013). Periscope endograft technique to revascularize the left subclavian artery during thoracic endovascular aortic repair. J. Endovasc. Ther..

[14] Pecoraro F., Lachat M., Hofmann M., Cayne N.S., Chaykovska L., Rancic Z., Puippe G., Pfammatter T., Mangialardi N., Veith F.J., Bettex D., Maisano F., Neff T.A. (2017). Mid-term results of zone 0 thoracic endovascular aneurysm repair after ascending aorta wrapping and supra-aortic debranching in high-risk patients. Interact. Cardiovasc. Thorac. Surg..

